# Supplementary material for: Splice-Junction-Based Mapping of Alternative Isoforms in the Human Proteome
Source: Cell Rep. Author manuscript; Available in PMC 2020 Jan 15. (PMC6961840; doi:10.1016/j.celrep.2019.11.026)
Supplement: 2 [file NIHMS1546469-supplement-2.zip › DF1/PXD006675/LeftAtrium/LeftAtrium_7_ART3_IPEPFPLPAPGPVPVPGPK.pdf]

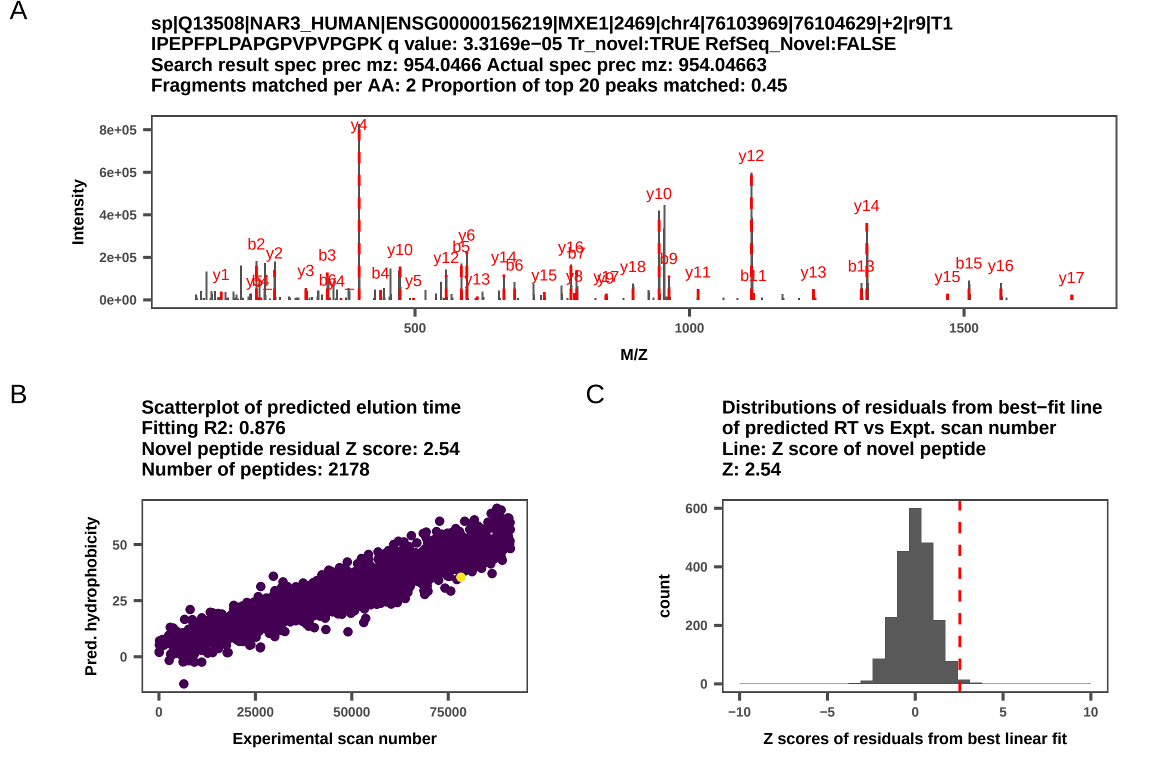
A
sp|Q13508|NAR3_HUMAN|ENSG00000156219|MXE1|2469|chr4|76103969|76104629|+2|r9|T1
IPEPFPLPAPGPVPVPGPK q value: 3.3169e−05 Tr_novel:TRUE RefSeq_Novel:FALSE
Search result spec prec mz: 954.0466 Actual spec prec mz: 954.04663
Fragments matched per AA: 2 Proportion of top 20 peaks matched: 0.45
Intensity
8e+05
6e+05
4e+05
2e+05
0e+00
y4
y12
y10
y14
b2
y2
y1
b3
y3
y10
y5
b4
y12
b5
y6
y13
b6
y15
y16
b7
y18
b9
y11
b11
y13
b13
y15
b15
y16
y17
500
1000
1500
M/Z
B
Scatterplot of predicted elution time
Fitting R2: 0.876
Novel peptide residual Z score: 2.54
Number of peptides: 2178
Pred. hydrophobicity
50
25
0
0
25000
50000
75000
Experimental scan number
C
Distributions of residuals from best−fit line
of predicted RT vs Expt. scan number
Line: Z score of novel peptide
Z: 2.54
count
600
400
200
0
−10
−5
0
5
10
Z scores of residuals from best linear fit
